# Glucose-to-albumin ratio and mortality in ICU patients with acute pancreatitis

**DOI:** 10.1515/med-2026-1511

**Published:** 2026-08-31

**Authors:** Qingqing Xiao, Siwen Wu

**Affiliations:** Department of Laboratory Medicine, The First Affiliated Hospital, Zhejiang University School of Medicine, Hangzhou, China; Department of Blood Transfusion, The First Affiliated Hospital, Zhejiang University School of Medicine, Hangzhou, China

**Keywords:** glucose-to-albumin ratio, acute pancreatitis, intensive care unit, mortality prediction, machine learning

## Abstract

**Objectives:**

Acute pancreatitis (AP) carries a high mortality risk in ICU patients. The glucose-to-albumin ratio (GAR), reflecting metabolic stress and albumin status, may serve as a simple prognostic marker. This study evaluated the association between GAR and mortality in ICU patients with AP.

**Methods:**

Data from 754 ICU patients with AP from the MIMIC-IV database were analyzed. Patients were divided into quartiles based on GAR. Multivariable Cox regression, Kaplan-Meier survival analysis, Boruta algorithm, and machine learning models were used to assess the relationship between GAR and 30-day and 90-day mortality.

**Results:**

Elevated GAR was independently associated with increased mortality risk. For 30-day mortality, GAR had the highest AUC (0.69) compared to albumin, SOFA, and glucose. Patients in the highest GAR quartile had a 10.2-fold increased risk of 30-day mortality (p<0.001). The Boruta algorithm identified GAR as an important predictor, and LightGBM showed the best performance (AUC=0.83).

**Conclusions:**

GAR was independently associated with 30-day and 90-day mortality in ICU patients with AP. As a simple and readily available biomarker, GAR may help improve early mortality risk stratification in this critically ill population.

## Introduction

Acute pancreatitis is a prevalent and potentially fatal condition, with its global incidence rising by nearly 60 % over the past two decades [1], [2], [3]. Although some cases of acute pancreatitis can be resolved with fluid resuscitation and supportive care [4], 5], approximately 20 % of patients progress to moderate or severe acute pancreatitis, often resulting in severe complications, prolonged hospital stays, and an overall mortality rate of 20–40 % [6], 7]. The persistently rising incidence and high mortality rate of acute pancreatitis further emphasize the urgent need for novel and reliable biomarkers to improve prognostic prediction, guide clinical decision-making, and enhance patient outcomes.

Traditional biomarkers, such as the lactate-to-albumin ratio [7], neutrophil-to-lymphocyte ratio [8], and platelet-to-lymphocyte ratio [9], have been commonly used to assess the inflammatory response in acute pancreatitis. However, these biomarkers are limited to reflecting only the inflammatory dimension of the disease, and fail to provide a comprehensive view of the patient’s overall condition. In contrast, the glucose-to-albumin ratio (GAR) has recently gained attention as a promising multidimensional biomarker, reflecting both glucose fluctuations and nutritional/inflammatory status [10]. GAR is not limited to inflammation but also integrates metabolic and nutritional factors, offering a more holistic perspective of the patient’s condition. Although GAR has demonstrated prognostic value in conditions such as cardiovascular diseases and postoperative infections [10], 11], its specific role in acute illnesses, particularly in ICU patients with acute pancreatitis, remains poorly understood. ICU patients often experience stress-induced hyperglycemia on the first day of admission [12], coupled with hypoalbuminemia due to excessive energy consumption by the body in response to severe illness, leading to significant nutritional depletion [13]. These metabolic and inflammatory changes suggest that GAR could serve as a valuable predictive tool in critically ill patients with acute pancreatitis.

However, existing studies on mortality in acute pancreatitis have primarily focused on general populations, overlooking the unique metabolic and inflammatory challenges faced by ICU-admitted acute pancreatitis patients. To address these gaps, this study leverages the Medical Information Mart for Intensive Care IV (MIMIC-IV) database, a large, publicly available critical care dataset, to evaluate the prognostic role of GAR in ICU patients with acute pancreatitis. This study aims to provide novel insights into the utility of GAR as a biomarker in this high-risk population, ultimately contributing to improved prognostic tools and personalized interventions in ICU settings.

## Materials and methods

### Research design

This study utilized the MIMIC-IV database (version 3.1), an open-access dataset with de-identified ICU records from Beth Israel Deaconess Medical Center spanning 2008 to 2022. It includes information on demographics, laboratory results, vital signs, treatments, and outcomes. Access to the database required completing a data use agreement and certification in human research ethics. The first author Qingqing Xiao (71755612) met these requirements and performed data extraction and preprocessing.

**Figure 1:**
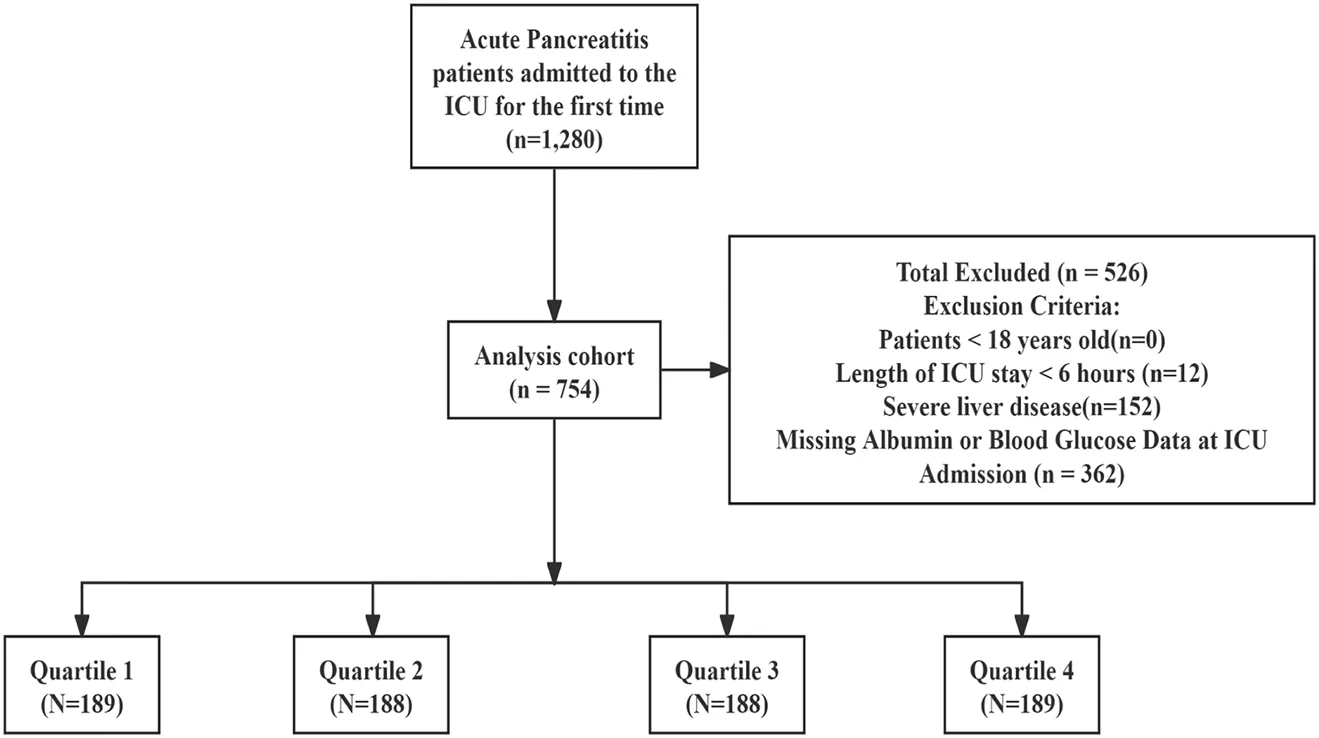
Study cohort inclusion flowchart.

A total of 1,280 patients with a primary diagnosis of acute pancreatitis admitted to the ICU for the first time were identified. After applying exclusion criteria, 526 patients were excluded: 0 were under 18 years old, 12 had ICU stays <6 h, 152 had severe liver disease, and 362 lacked albumin or glucose data at admission. The final cohort included 754 patients, divided into quartiles based on GAR levels: Quartile 1 (n=189), Quartile 2 (n=188), Quartile 3 (n=188), and Quartile 4 (n=189) (Figure 1).

### Data collection

Data for this study were extracted from the MIMIC-IV database (version 3.1) using PostgreSQL software. Only data from the first 24 h after ICU admission were included, and for variables measured multiple times within the first day, the average value was used. The collected data included demographics such as age, gender, and race; clinical scores including Sequential Organ Failure Assessment (SOFA), Acute Physiology Score III (APS III), Oxford Acute Severity of Illness Score (OASIS), Glasgow Coma Scale (GCS), and Charlson Comorbidity Index (CCI); vital signs including heart rate, respiratory rate, temperature, systolic blood pressure (SBP), and oxygen saturation (SpO_2_); comorbidities including chronic pulmonary disease, malignant cancer, diabetes, hypertension, acute kidney injury (AKI), and sepsis; laboratory tests including red blood cell count (RBC), white blood cell count (WBC), platelet count, sodium, potassium, calcium, glucose, albumin, alanine aminotransferase (ALT), aspartate aminotransferase (AST), prothrombin time, international normalized ratio (INR), anion gap, blood urea nitrogen (BUN), and creatinine; treatments including endoscopic retrograde cholangiopancreatography (ERCP), norepinephrine, dopamine, octreotide, mechanical ventilation, and continuous renal replacement therapy (CRRT); and outcomes such as hospital mortality, ICU mortality, hospital and ICU stay durations, as well as 30-day and 90-day hospital mortality.

### Definition and clinical results

The first available serum glucose and serum albumin values within 24 h after ICU admission were extracted for GAR calculation. GAR was calculated as serum glucose converted from mg/dL to mmol/L by dividing by 18, divided by serum albumin (g/dL) [14]. The main clinical outcome was 30-day in-hospital mortality, referring to all-cause deaths within 30 days of hospitalization. The secondary outcome was 90-day in-hospital mortality, defined as all-cause deaths within 90 days of hospitalization.

### Statistical analysis

#### Methods for association analysis between GAR and mortality in ICU patients with AP

Continuous variables were presented as mean ± standard deviation or median with interquartile range according to their distribution, and categorical variables as numbers and percentages. Normality was assessed using the Kolmogorov-Smirnov test. Comparisons between groups were performed using Student’s *t*-test, ANOVA, Mann-Whitney U test, Kruskal-Wallis test, chi-square test, or Fisher’s exact test, as appropriate. Variables with more than 10 % missing values were excluded, and multiple imputation was applied to variables with less than 10 % missingness. The proportions of missing data are presented in Supplementary Material (Table S1).

Kaplan-Meier curves and log-rank tests were used to compare 30-day and 90-day mortality across GAR quartiles. Cox proportional hazards models were used to evaluate GAR both as a continuous variable and as quartiles, with Quartile 1 as the reference. Model 1 was unadjusted; Model 2 was adjusted for age, sex, and race; and Model 3 was further adjusted for SOFA, CCI, SBP, RBC, ALT, AST, anion gap, malignant cancer, diabetes, hypertension, sepsis, ERCP, norepinephrine, octreotide, mechanical ventilation, and CRRT. The proportional hazards assumption was tested using Schoenfeld residuals, and hazard ratios with 95 % confidence intervals were reported.

Restricted cubic spline (RCS) regression with three knots was used to assess potential non-linear associations between GAR and mortality. Subgroup analyses were conducted according to age, gender, race, chronic pulmonary disease, malignant cancer, diabetes, sepsis, and mechanical ventilation. Receiver operating characteristic (ROC) curves were used to compare the predictive performance of GAR with other variables.

#### Methods for machine learning-based mortality prediction

The machine learning analysis was based on baseline clinical characteristics. Treatment-related variables, including liquid balance, dextrose-containing fluids, albumin infusion, and corticosteroid use, were not included in the feature-selection process because they may reflect early ICU management rather than baseline patient status and were therefore addressed separately in sensitivity analyses. The Boruta algorithm was used to identify important predictors of 30-day mortality. The dataset was randomly divided into a training set and a validation set at a ratio of 7:3 using stratified sampling according to 30-day mortality status. Variables selected by the Boruta algorithm were then used to construct multiple machine learning models, including Random Forest (RF), Extreme Gradient Boosting (XGBoost), Light Gradient Boosting Machine (LightGBM), k-Nearest Neighbors (KNN), Support Vector Machine (SVM), and Multilayer Perceptron (MLP). Bayesian optimization was performed to tune the hyperparameters of each model.

Model performance was evaluated in the validation set using the area under the receiver operating characteristic curve (AUC), decision curve analysis, accuracy, sensitivity, specificity, Kappa coefficient, Matthews correlation coefficient, F-score, and the area under the precision-recall curve. The best-performing model was further interpreted using SHapley Additive exPlanations (SHAP) to assess the contribution of each predictor to model predictions.

#### Methods for sensitivity analysis of the GAR–mortality association GAR

Sensitivity analyses were conducted to assess the robustness of the main findings. Model 3 was regarded as the main analysis. Cox regression analyses were repeated after excluding patients who received insulin therapy, dextrose-containing fluids, albumin infusion, or corticosteroid use. Additional adjustment for liquid balance and AKI was also performed in the fully adjusted model. Finally, log-transformed GAR was analyzed as a continuous variable to evaluate the consistency of the association. Hazard ratios and 95 % confidence intervals were reported. In addition, baseline characteristics were compared between excluded and included patients to evaluate potential differences between these populations.

All analyses used R software (version 4.4.2), with significance set at a two-sided p<0.05.

## Results

### Baseline characteristics

**Figure 2:**
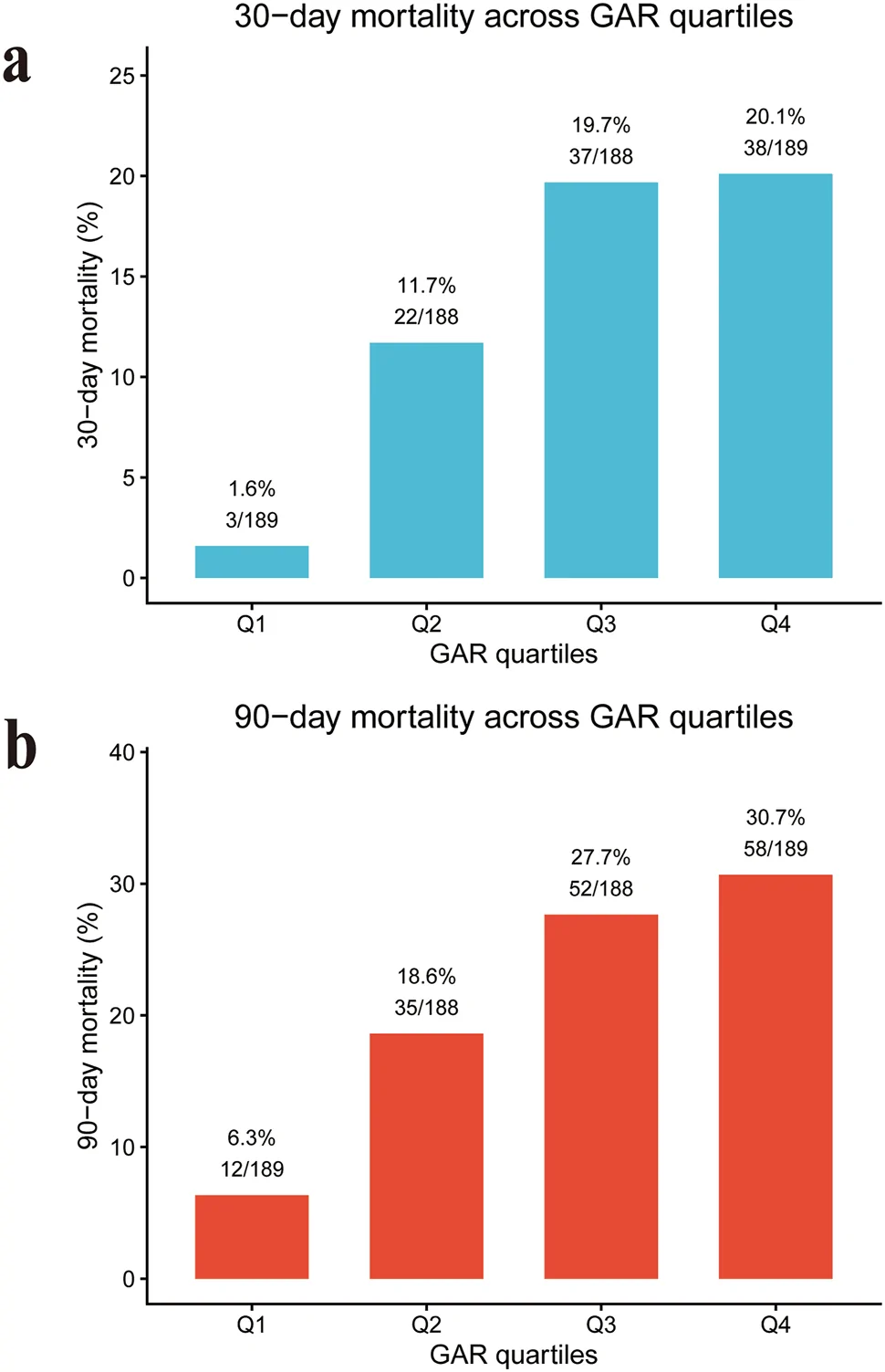
Number and proportion of deaths across GAR quartiles for 30-day (a) and 90-day, (b) all-cause mortality in patients with acute pancreatitis.

Table 1 provides an overview of baseline characteristics grouped by GAR quartiles. Significant differences were observed in race (p=0.036), SOFA score (p<0.001), APS III (p<0.001), OASIS (p<0.001), heart rate (p<0.001), respiratory rate (p<0.001), SBP (p=0.005), chronic pulmonary disease (p=0.029), diabetes (p<0.001), AKI (p<0.001), sepsis (p=0.007), RBC (p=0.010), WBC (p<0.001), platelet count (p=0.012), sodium (p=0.022), potassium (p=0.024), calcium (p<0.001), glucose (p<0.001), albumin (p<0.001), PT (p<0.001), INR (p<0.001), BUN (p<0.001), ERCP (p=0.016), norepinephrine (p<0.001), mechanical ventilation (p<0.001), CRRT (p<0.001), hospital mortality (p<0.001), ICU mortality (p<0.001), hospital stay duration (p<0.001), ICU stay duration (p<0.001), 30-day mortality (p<0.001), and 90-day mortality (p<0.001). The numbers of deaths across GAR quartiles are presented in Figure 2.

**Table 1:** Baseline characteristics stratified by GAR quartiles.

Characteristic	Overall n=754	Quartile 1 n=189	Quartile 2 n=188	Quartile 3 n=188	Quartile 4 n=189	p-Value
**Demographics**						
Age, years	60.34 (47.47, 74.06)	61.24 (45.59, 75.85)	60.97 (48.24, 75.63)	58.04 (47.33, 72.18)	61.49 (48.60, 73.83)	0.561
Gender, male (%)	427 (57 %)	112 (59 %)	100 (53 %)	102 (54 %)	113 (60 %)	0.451
Race, n (%)						**0.036**
White	508 (67.37)	132 (69.84)	142 (75.53)	115 (61.17)	119 (62.96)	
Black	72 (9.55)	15 (7.94)	18 (9.57)	19 (10.11)	20 (10.58)	
Other	174 (23.08)	42 (22.22)	28 (14.89)	54 (28.72)	50 (26.46)	
**Clinical scores**						
SOFA	1.00 (0.00, 3.00)	1.00 (0.00, 2.00)	1.00 (0.00, 3.00)	1.00 (0.00, 3.00)	2.00 (0.00, 4.00)	**<0.001**
APS III	48.00 (36.00, 68.00)	39.00 (29.00, 48.00)	46.00 (36.50, 60.50)	50.50 (38.00, 75.00)	63.00 (46.00, 85.00)	**<0.001**
OASIS	33.00 (26.00, 40.00)	29.00 (24.00, 34.00)	31.00 (26.00, 37.00)	35.00 (28.00, 42.00)	37.00 (31.00, 44.00)	**<0.001**
GCS	15.00 (15.00, 15.00)	15.00 (15.00, 15.00)	15.00 (15.00, 15.00)	15.00 (15.00, 15.00)	15.00 (15.00, 15.00)	0.645
CCI	3.00 (2.00, 6.00)	3.00 (1.00, 5.00)	3.00 (2.00, 6.00)	3.00 (1.00, 6.00)	4.00 (2.00, 6.00)	0.104
**Vital signs**						
Heart rate, bpm	95.00 (83.22, 107.38)	86.73 (77.13, 101.19)	94.28 (80.72, 106.10)	98.42 (86.86, 109.03)	98.00 (89.84, 111.89)	**<0.001**
Respiratory rate, bpm	20.88 (17.79, 24.16)	18.74 (16.62, 21.93)	20.45 (17.24, 23.23)	21.79 (18.67, 24.69)	21.90 (19.11, 25.33)	**<0.001**
Temperature, °C	36.97 (36.69, 37.35)	36.92 (36.68, 37.18)	36.96 (36.66, 37.33)	37.06 (36.75, 37.48)	36.99 (36.69, 37.41)	0.088
SBP, mmHg	119.02 (108.51, 134.17)	122.95 (111.94, 134.94)	118.49 (109.85, 136.72)	115.30 (106.27, 136.07)	115.58 (107.17, 129.32)	**0.005**
SpO_2_, %	96.11 (94.85, 97.43)	95.97 (94.85, 97.26)	96.07 (94.93, 97.16)	96.18 (94.93, 97.56)	96.24 (94.79, 97.52)	0.856
**Comorbidities, %**						
Chronic pulmonary disease, n (%)	157 (21 %)	53 (28 %)	39 (21 %)	31 (16 %)	34 (18 %)	**0.029**
Malignant cancer, n (%)	68 (9 %)	12 (6 %)	18 (10 %)	18 (10 %)	20 (11 %)	0.504
Diabetes, n (%)	241 (32 %)	30 (16 %)	44 (23 %)	67 (36 %)	100 (53 %)	**<0.001**
Hypertension, n (%)	253 (34 %)	72 (38 %)	62 (33 %)	68 (36 %)	51 (27 %)	**0.111**
AKI, n (%)	525 (70 %)	101 (53 %)	133 (71 %)	144 (77 %)	147 (78 %)	**<0.001**
Sepsis, n (%)	476 (63 %)	102 (54 %)	118 (63 %)	122 (65 %)	134 (71 %)	**0.007**
**Laboratory test**						
RBC, 10^9^/L	3.55 (3.12, 4.05)	3.69 (3.30, 4.14)	3.58 (3.15, 4.14)	3.50 (3.11, 4.01)	3.43 (2.94, 3.96)	**0.010**
WBC, 10^9^/L	12.60 (8.70, 17.80)	10.10 (7.30, 14.50)	12.00 (8.58, 16.80)	14.46 (10.03, 19.90)	14.15 (10.83, 21.25)	**<0.001**
Platelet, 10^9^/L	185.00 (128.00, 260.00)	169.33 (114.25, 229.00)	184.50 (135.58, 256.63)	201.13 (134.25, 297.90)	186.50 (123.25, 275.33)	**0.012**
Sodium, mmol/L	138.00 (135.20, 140.75)	138.67 (136.00, 140.67)	138.71 (135.50, 141.00)	137.53 (135.23, 140.45)	136.83 (133.80, 141.25)	**0.022**
Potassium, mmol/L	4.00 (3.70, 4.39)	3.94 (3.65, 4.30)	3.95 (3.72, 4.30)	4.06 (3.75, 4.37)	4.08 (3.67, 4.60)	**0.024**
Calcium, mg/dL	7.93 (7.45, 8.45)	8.20 (7.83, 8.60)	8.05 (7.50, 8.49)	7.70 (7.27, 8.25)	7.70 (7.27, 8.20)	**<0.001**
Glucose, mg/dL	128.73 (106.50, 163.52)	101.57 (92.13, 111.43)	121.06 (106.11, 133.98)	141.74 (123.45, 164.09)	189.50 (158.07, 227.56)	**<0.001**
Albumin, g/dL	3.00 (2.50, 3.40)	3.50 (3.10, 3.90)	3.00 (2.75, 3.40)	2.75 (2.40, 3.20)	2.40 (2.10, 2.90)	**<0.001**
GAR	25.22 (19.18, 33.77)	16.52 (14.70, 17.93)	22.19 (20.45, 23.52)	28.74 (26.85, 31.01)	42.49 (37.03, 48.73)	**<0.001**
ALT, U/L	48.88 (24.00, 137.00)	66.67 (30.00, 170.50)	50.50 (25.00, 143.63)	46.58 (24.50, 140.00)	39.67 (20.00, 89.00)	**0.012**
AST, U/L	69.00 (34.50, 150.33)	81.00 (39.00, 164.00)	62.75 (34.83, 173.08)	66.67 (34.50, 141.38)	62.00 (30.00, 129.50)	0.173
PT, S	14.23 (12.93, 16.45)	13.60 (12.30, 15.55)	14.22 (13.03, 15.92)	14.83 (13.15, 17.18)	14.57 (13.20, 17.23)	**<0.001**
INR	1.30 (1.15, 1.50)	1.20 (1.10, 1.40)	1.30 (1.19, 1.44)	1.34 (1.20, 1.59)	1.33 (1.20, 1.60)	**<0.001**
Anion gap, m q/L	14.50 (12.25, 17.00)	14.33 (12.33, 16.50)	14.21 (12.33, 16.75)	14.50 (12.00, 17.00)	14.75 (12.00, 17.75)	0.646
BUN, mg/dL	19.17 (11.67, 35.50)	15.00 (9.00, 25.67)	18.17 (11.75, 32.00)	19.00 (12.08, 35.11)	26.83 (15.29, 43.75)	**<0.001**
Creatinine, mg/dL	1.00 (0.70, 1.90)	0.90 (0.68, 1.37)	1.00 (0.72, 1.85)	0.96 (0.67, 1.83)	1.32 (0.75, 2.60)	**0.003**
Liquid balance, mL	3,857.72 (1,316.06, 6,122.88)	2,350.34 (655.00, 4,257.00)	3,611.89 (1,292.04, 5,722.68)	4,256.50 (1,871.83, 6,976.11)	4,258.00 (1,795.17, 8,828.05)	**<0.001**
**Treatments**						
ERCP, %	34 (5 %)	6 (3 %)	16 (9 %)	4 (2 %)	8 (4 %)	**0.016**
Norepinephrine, %	198 (26 %)	23 (12 %)	39 (21 %)	64 (34 %)	72 (38 %)	**<0.001**
Dopamine, %	20 (3 %)	2 (1 %)	5 (3 %)	7 (4 %)	6 (3 %)	0.371
Octreotide, %	35 (5 %)	5 (3 %)	6 (3 %)	9 (5 %)	15 (8 %)	0.065
Mechanical ventilation, %	587 (78 %)	120 (63 %)	151 (80 %)	156 (83 %)	160 (85 %)	**<0.001**
CRRT, %	87 (12 %)	5 (3 %)	12 (6 %)	30 (16 %)	40 (21 %)	**<0.001**
Albumin infusion, n (%)	276 (36.60)	52 (27.51)	72 (38.30)	71 (37.77)	81 (42.86)	**0.017**
Dextrose fluid, n (%)	536 (71.09)	121 (64.02)	142 (75.53)	134 (71.28)	139 (73.54)	0.073
Corticosteroid, n (%)	16 (2.12)	2 (1.06)	6 (3.19)	3 (1.60)	5 (2.65)	0.454
Insulin, n (%)	351 (46.55)	58 (30.69)	80 (42.55)	89 (47.34)	124 (65.61)	**<0.001**
**Events**						
Hospital mortality, %	108 (14 %)	2 (1 %)	20 (11 %)	40 (21 %)	46 (24 %)	**<0.001**
ICU mortality, %	70 (9 %)	2 (1 %)	11 (6 %)	24 (13 %)	33 (17 %)	**<0.001**
Los of hospital, day	12.59 (6.59, 21.96)	6.89 (4.70, 11.85)	12.08 (6.62, 18.47)	15.76 (8.79, 26.62)	18.70 (9.75, 30.49)	**<0.001**
Los of ICU, day	2.85 (1.42, 7.02)	1.82 (1.04, 3.11)	2.55 (1.35, 5.92)	3.50 (1.81, 9.70)	4.60 (1.86, 12.70)	**<0.001**
30-day hospital mortality, %	100 (13 %)	3 (2 %)	22 (12 %)	37 (20 %)	38 (20 %)	**<0.001**
90-day hospital mortality, %	157 (21 %)	12 (6 %)	35 (19 %)	52 (28 %)	58 (31 %)	**<0.001**

GAR, quartile 1 (10.51–19.18), quartile 2 (19.18–25.22), quartile 3 (25.22–33.77), quartile 4 (33.77–179.17); GAR, glucose-to-albumin ratio; SBP, systolic blood pressure; WBC, white blood cell count; RBC, red blood cell count; Platelet, platelet count; SOFA, Sequential Organ Failure Assessment; GCS, Glasgow Coma Scale; APS III, Acute Physiology Score III; OASIS, Oxford Acute Severity of Illness Score; SpO_2_, oxygen saturation; CCI, Charlson Comorbidity Index; AKI, acute kidney injury; INR, international normalized ratio; BUN, blood urea nitrogen; AST, aspartate aminotransferase; ALT, alanine aminotransferase; ERCP, endoscopic retrograde cholangiopancreatography; CRRT, continuous renal replacement therapy. Bold values indicate statistical significance (p<0.05).

### Primary results

**Figure 3:**
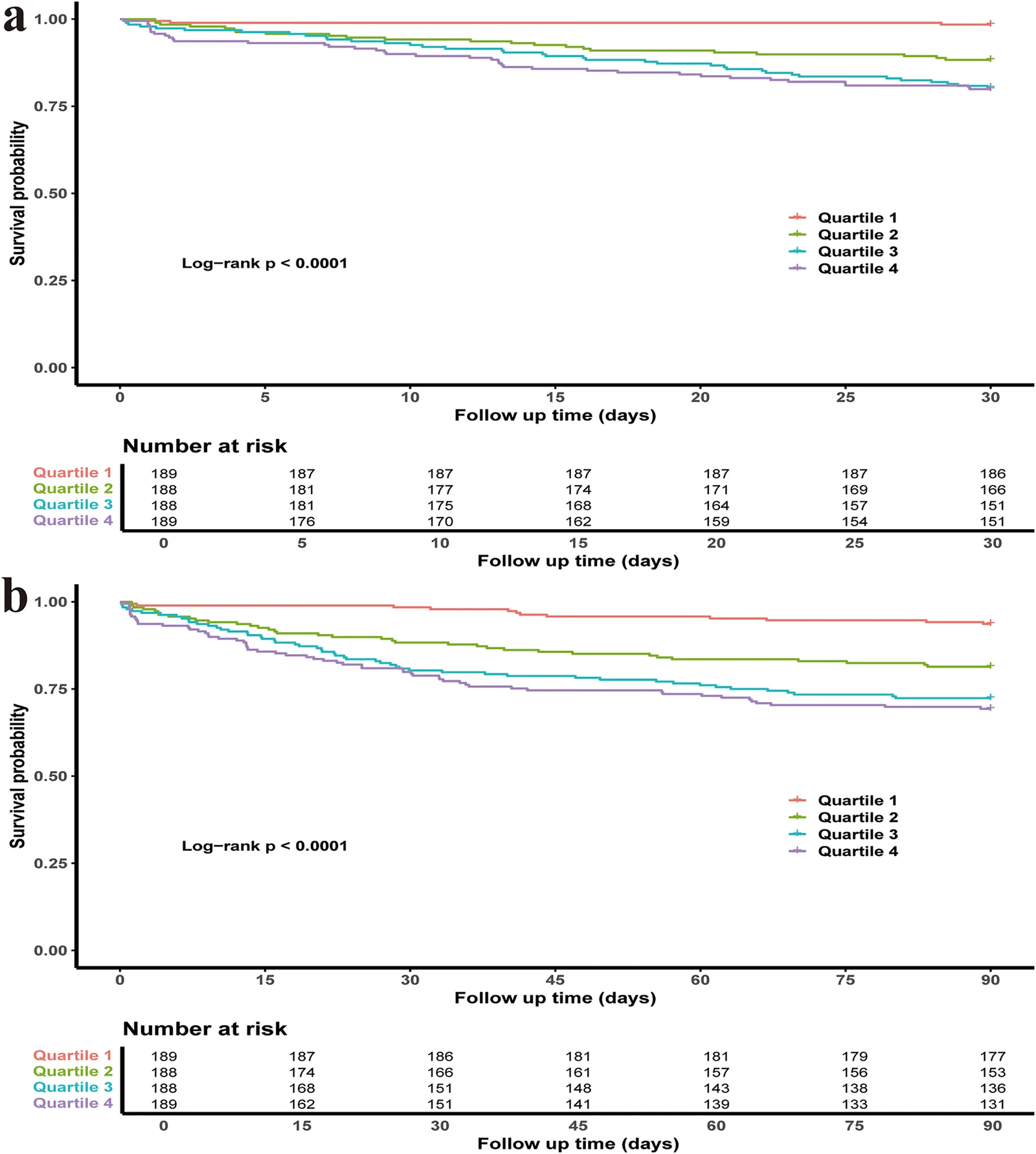
Kaplan-Meier curves for 30-day (a) and 90-day, (b) all-cause mortality in acute pancreatitis patients stratified by GAR quartiles.

The Kaplan-Meier survival curves stratified by GAR quartiles show significant differences in 30-day and 90-day survival probabilities (log-rank p<0.0001), with Quartile 4 having the lowest survival (Figure 3).

Variables included in the multivariable Cox regression models were selected using a combined statistical and clinical approach. Candidate covariates were initially identified based on univariable Cox regression analysis, with the corresponding results presented in the Supplementary Material (Table S2), and the Boruta algorithm (Figure 6). To avoid overfitting, multicollinearity, and overadjustment, the final covariate set was further refined according to clinical relevance and recommendations from clinical experts, and clinically important variables were considered even if they were not selected solely by statistical criteria. Table 2 displays the results of the multivariable Cox regression. In the fully adjusted Model 3, GAR as a continuous variable had an HR of 1.02 (95 % CI: 1.01–1.03, p=0.002) for 30-day all-cause mortality. Compared to Quartile 1, Quartile 4 was associated with a higher risk of 30-day all-cause mortality (HR=10.2, 95 % CI: 3.00–34.4, p<0.001), with a significant trend across quartiles (p for trend<0.001). Similarly, for 90-day mortality, GAR as a continuous variable showed an HR of 1.02 (95 % CI: 1.01–1.03, p<0.001), while Quartile 4 was associated with a higher risk of 90-day mortality compared with Quartile 1 (HR=4.62, 95 % CI: 2.38–8.99, p<0.001), again showing a significant trend (p for trend<0.001). The proportional hazards assumption was assessed using Schoenfeld residuals, and no significant violation was observed for the Cox models (all p>0.05; Figure S1, Supplementary Material).

**Table 2:** Multivariable cox regression analysis of GAR and all-cause mortality.

Variables	Model 1		Model 2		Model 3	
	HR (95 % CI)	p-Value	HR (95 % CI)	p-Value	HR (95 % CI)	p-Value
**30-day**						
Continuous variable	1.02 (1.01, 1.03)	**<0.001**	1.02 (1.01, 1.02)	**<0.001**	1.02 (1.01, 1.03)	**0.002**
GAR quartiles						
Quartile 1	–		–		–	
Quartile 2	7.77 (2.33, 26.0)	**<0.001**	8.48 (2.54, 28.4)	**<0.001**	7.56 (2.54, 28.4)	**0.001**
Quartile 3	13.5 (4.16, 43.7)	**<0.001**	16.2 (4.97, 52.8)	**<0.001**	12.0 (3.60, 40.3)	**<0.001**
Quartile 4	14.1 (4.36, 45.8)	**<0.001**	15.7 (4.83, 51.0)	**<0.001**	10.2 (3.00, 34.4)	**<0.001**
**P for trend**		**<0.001**		**<0.001**		**<0.001**
**90-day**						
Continuous variable	1.02 (1.02, 1.03)	**<0.001**	1.02 (1.01, 1.02)	**<0.001**	1.02 (1.01, 1.03)	**<0.001**
GAR quartiles						
Quartile 1	–		–		–	
Quartile 2	3.18 (1.65, 6.12)	**<0.001**	3.37 (1.74, 6.50)	**<0.001**	3.19 (1.63, 6.23)	**<0.001**
Quartile 3	4.99 (2.66, 9.35)	**<0.001**	6.05 (3.21, 11.4)	**<0.001**	5.12 (2.66, 9.86)	**<0.001**
Quartile 4	5.71 (3.07, 10.6)	**<0.001**	6.44 (3.45, 12.0)	**<0.001**	4.62 (2.38, 8.99)	**<0.001**
**P for trend**		**<0.001**		**<0.001**		**<0.001**

Model 1, crude; model 2, adjusted for age, gender, race; model 3, adjusted for model 2 + SOFA + CCI + SBP + RBC + ALT + AST + anion gap + malignant cancer + diabetes + hypertension + sepsis + ERCP + norepinephrine + octreotide + mechanical ventilation + CRRT; GAR, quartile 1 (10.51–19.18), quartile 2 (19.18–25.22), quartile 3 (25.22–33.77), quartile 4 (33.77–179.17); GAR, glucose-to-albumin ratio; SOFA, Sequential Organ Failure Assessment; CCI, Charlson Comorbidity Index; RBC, red blood cell count; SBP, systolic blood pressure; ALT, alanine aminotransferase; AST, aspartate aminotransferase; ERCP, endoscopic retrograde cholangiopancreatography; CRRT, continuous renal replacement therapy. Bold values indicate statistical significance (p<0.05).

**Figure 4:**
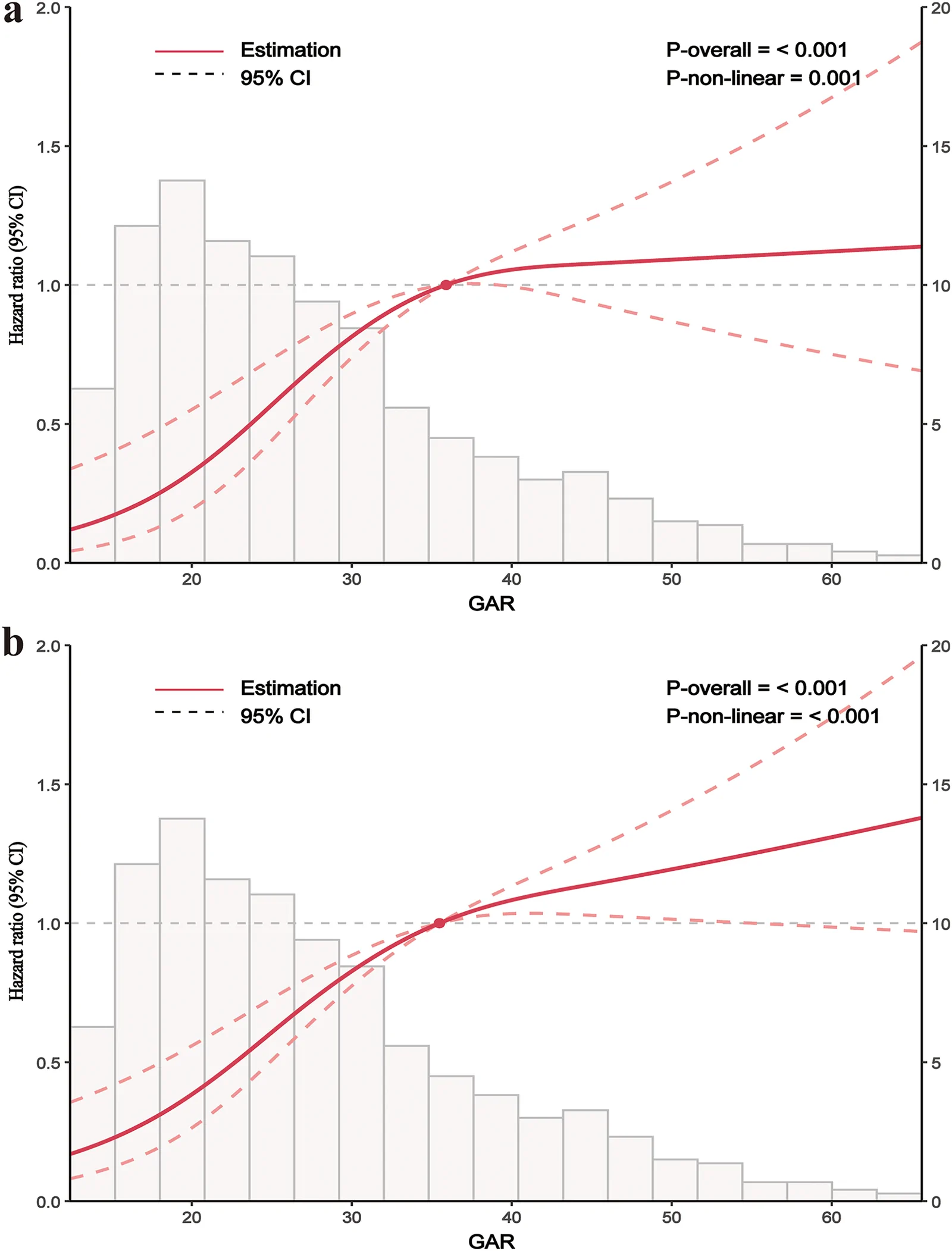
RCS analysis of GAR and all-cause mortality in patients with acute pancreatitis (a) 30-day mortality, (b) 90-day mortality.

The RCS analysis demonstrates a nonlinear relationship between GAR and mortality for both 30-day and 90-day outcomes (Figure 4). For 30-day mortality, the overall association is significant (P-overall <0.001), with evidence of non-linearity (P-non-linear=0.001). For 90-day mortality, the overall association is also significant (P-overall <0.001), with non-linearity observed (P-non-linear <0.001).

Table 3 presents the AUC values for various biomarkers and scores in predicting mortality at different time points in ICU patients with acute pancreatitis. The GAR consistently demonstrates superior predictive performance across all time points (30-day, 90-day, ICU, and hospital mortality) when compared to other markers. Specifically, GAR shows the highest AUC for ICU mortality (0.72) and hospital mortality (0.72), as well as for 30-day (0.69) and 90-day (0.67) mortality, surpassing traditional biomarkers such as albumin, glucose, and SOFA (Figure S2 Supplementary Material).

**Table 3:** Mortality prediction performance in ICU patients with acute pancreatitis.

	AUC (95 % CI)	Accuracy	Sensitivity	Specificity	PPV	NPV	Cut off
30-day hospital mortality							
GAR	0.69 (0.64–0.73)	0.53	0.83	0.49	0.20	0.95	23.822
Albumin	0.66 (0.59–0.71)	0.70	0.58	0.72	0.24	0.92	2.6
Glucose	0.61 (0.55–0.66)	0.53	0.68	0.51	0.17	0.91	127.5
SOFA	0.65 (0.59–0.71)	0.69	0.52	0.71	0.22	0.91	3
90-day hospital mortality							
GAR	0.67 (0.63–0.71)	0.57	0.78	0.51	0.29	0.90	24.141
Albumin	0.65 (0.60–0.70)	0.69	0.53	0.73	0.34	0.85	2.6
Glucose	0.59 (0.54–0.64)	0.52	0.68	0.48	0.25	0.85	123.625
SOFA	0.66 (0.61–0.70)	0.63	0.62	0.63	0.31	0.87	2
Hospital mortality							
GAR	0.72 (0.67–0.76)	0.55	0.87	0.50	0.22	0.96	23.822
Albumin	0.66 (0.60–0.72)	0.71	0.60	0.72	0.27	0.92	2.6
Glucose	0.64 (0.58–0.69)	0.54	0.74	0.51	0.20	0.92	126
SOFA	0.67 (0.62–0.73)	0.63	0.66	0.62	0.22	0.92	2
ICU mortality							
GAR	0.72 (0.66–0.77)	0.52	0.87	0.49	0.15	0.97	24.153
Albumin	0.65 (0.56–0.72)	0.69	0.59	0.70	0.17	0.94	2.6
Glucose	0.64 (0.57–0.71)	0.65	0.63	0.66	0.16	0.95	144.9
SOFA	0.69 (0.62–0.76)	0.70	0.60	0.71	0.18	0.95	3

### Subgroup analysis

**Figure 5:**
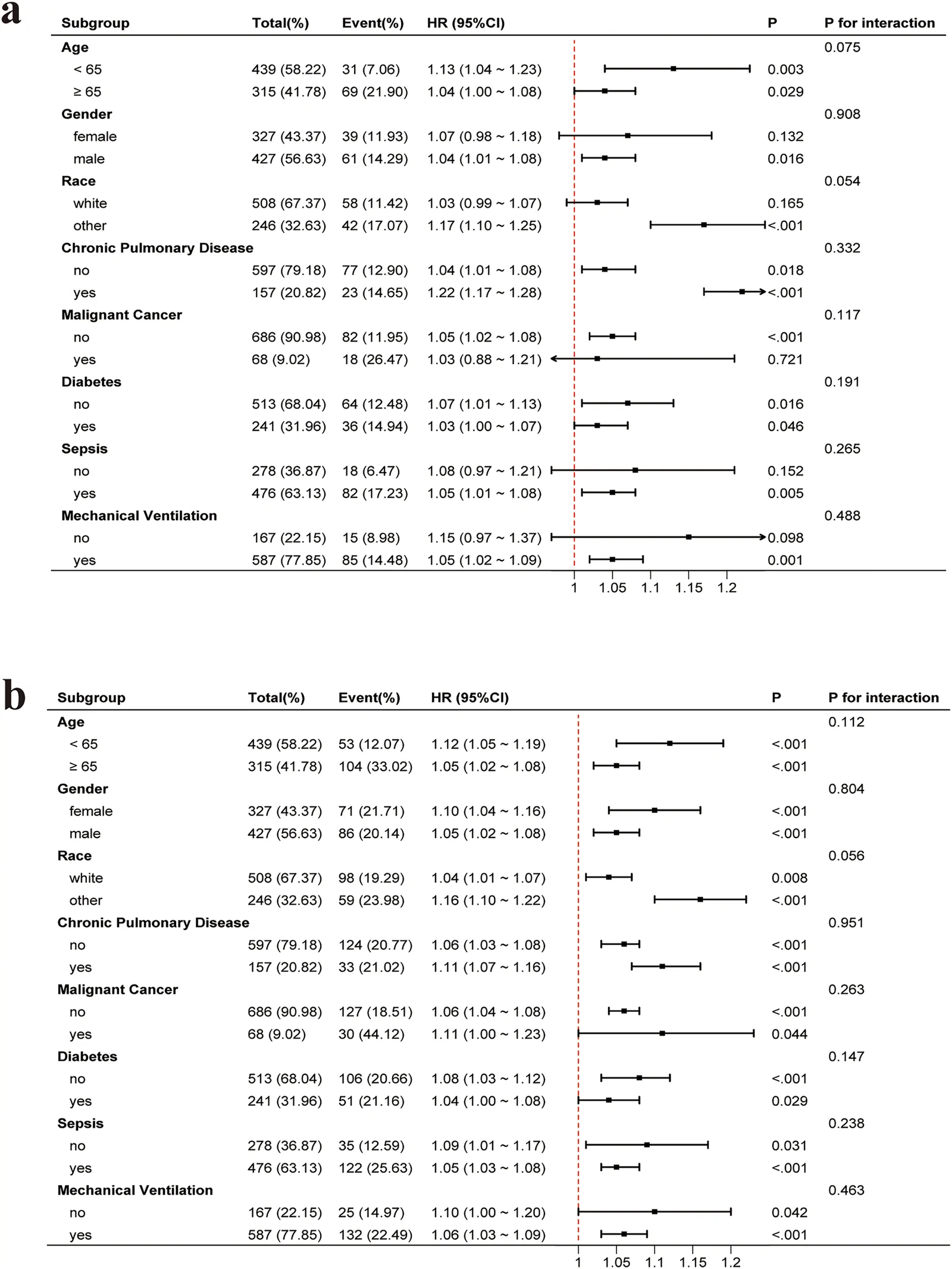
Subgroup forest plot of adjusted covariates for mortality in patients with acute pancreatitis (a) 30-day all-cause mortality, (b) 90-day all-cause mortality.

After adjusting for covariates, interaction tests were performed across predefined subgroups. No significant interactions were observed in any of the subgroups (Figure 5).

### Boruta

**Figure 6:**
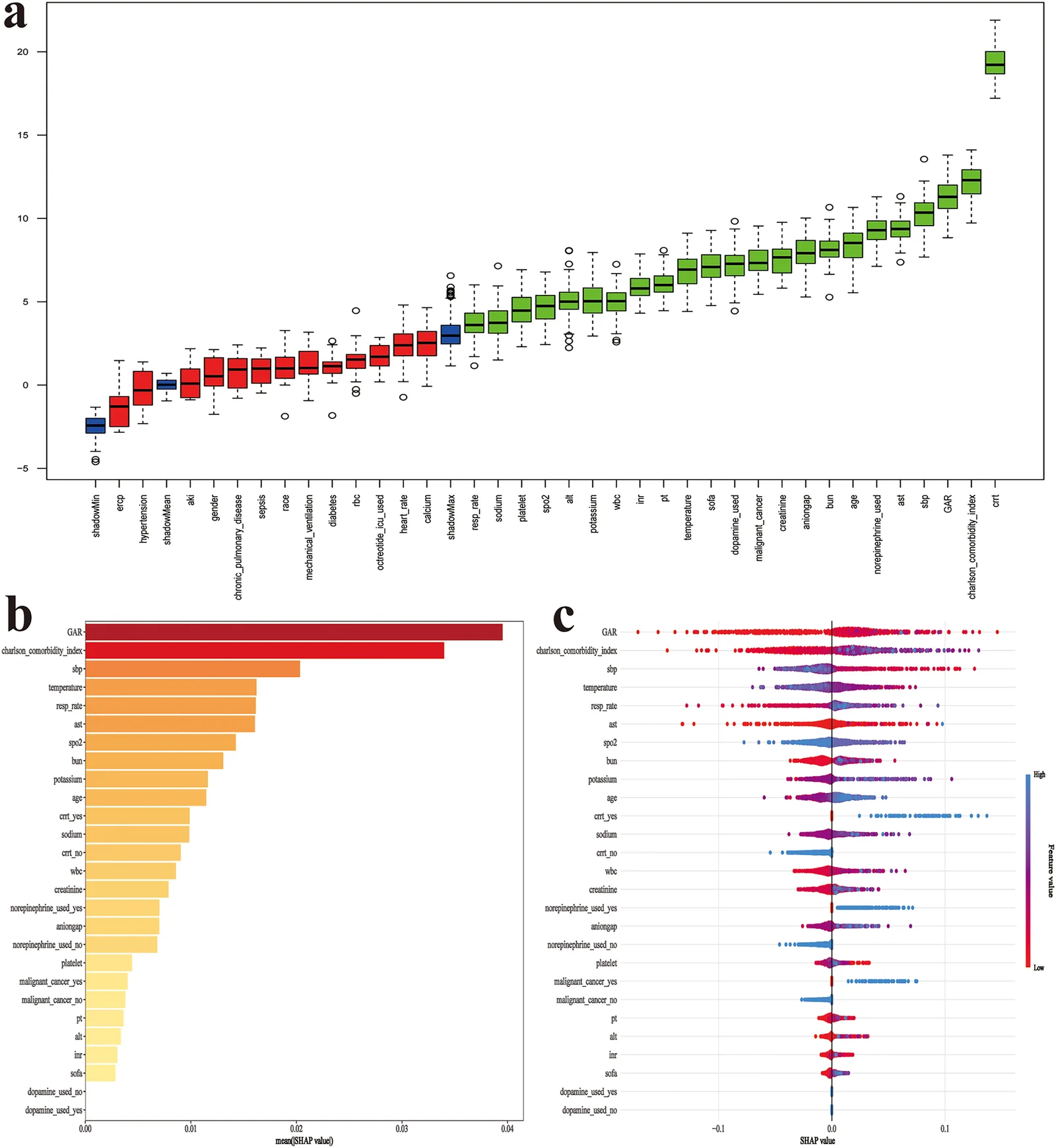
Feature selection and model interpretation for 30-day mortality prediction. (a) Boruta algorithm identifying key variables for predicting 30-day mortality (green indicates important variables), (b) SHAP-based variable importance ranking in the LightGBM model, (c) SHAP summary plot for variable importance in the LightGBM model.

The Boruta algorithm was applied with 30-day mortality as the outcome (p-value threshold=0.05, max runs=1,000). In the plot (Figure 6), green bars represent variables identified as important, while red bars indicate variables deemed unimportant. Among all laboratory tests and vital sign variables, the GAR was identified as the most important variable. GAR was also ranked as the third most important variable overall, surpassing SOFA and many other clinical parameters.

### Establishment and validation of the prediction model

**Figure 7:**
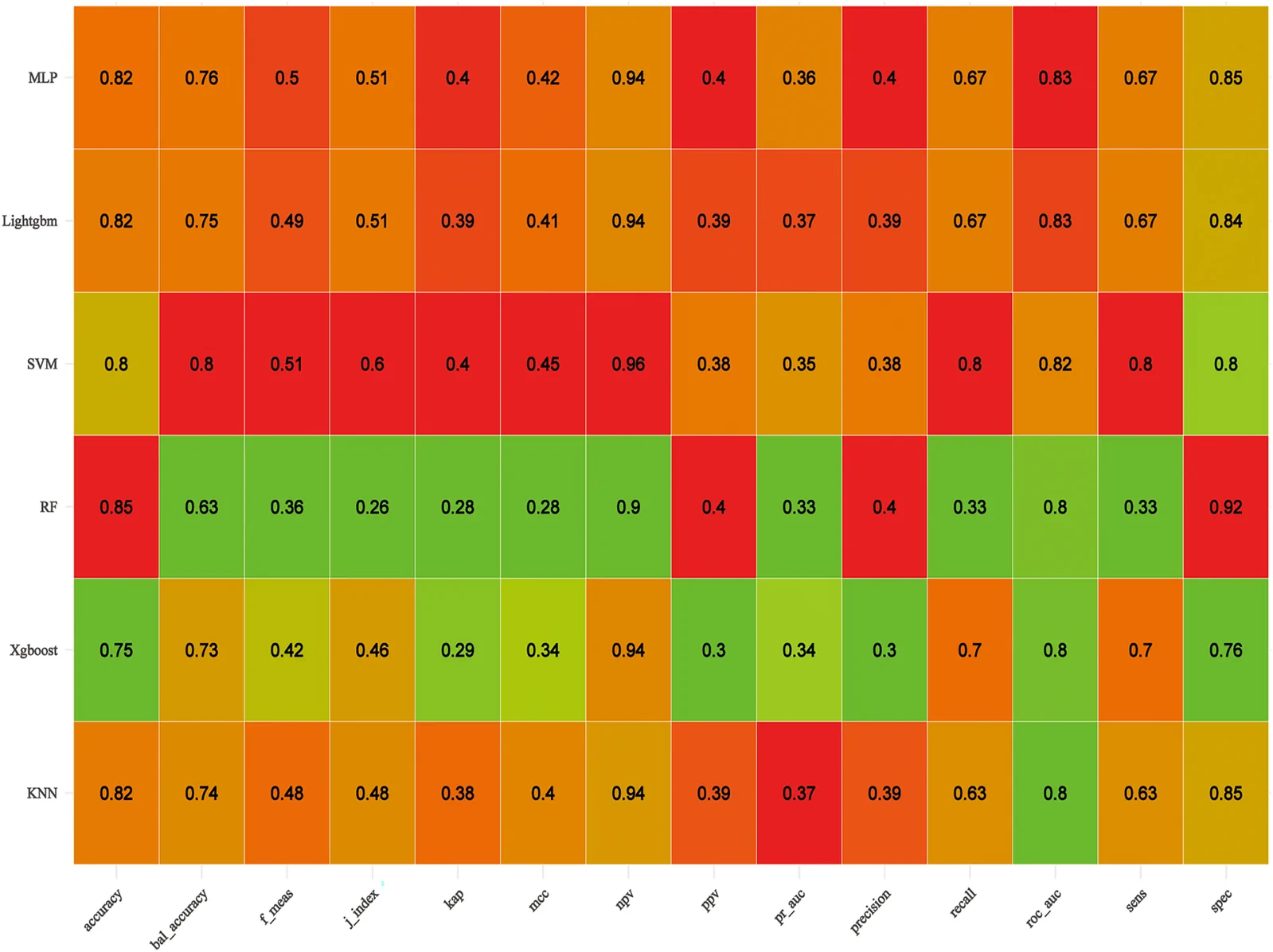
The comprehensive evaluation metrics of multiple models in the validation set, J index: Youden’s index, Kap: Cohen’s Kappa, MCC: Matthews correlation coefficient, NPV: negative predictive value, PPV: positive predictive value, PR auc: area under the precision-recall curve, roc auc: area under the receiver operating characteristic curve, sens: sensitivity, spec: specificity.

The original dataset was divided into a training set and a validation set according to a predefined protocol. Baseline characteristics were comparable between the two groups, with no significant differences observed in any variables; the corresponding results are presented in the Supplementary Material (Table S3). Additionally, the 30-day in-hospital mortality rates were 13 % in both the training and validation sets, indicating balanced sample characteristics after splitting. Machine learning models for predicting 30-day mortality were developed on the training set using variables identified as important (green) by the Boruta algorithm, and their performance was evaluated on the validation set. We trained six machine learning models, including RF, XGBoost, LightGBM, KNN, SVM, and MLP. Based on calibration curves and decision curve analysis, with the corresponding results presented in the Supplementary Material (Figure S3), as well as comprehensive evaluation metrics (Figure 7), LightGBM was identified as the best-performing model (AUC=0.83). SHAP analysis of the LightGBM model showed that GAR was the most important variable (Figure 7).

### Nomogram construction and validation

**Figure 8:**
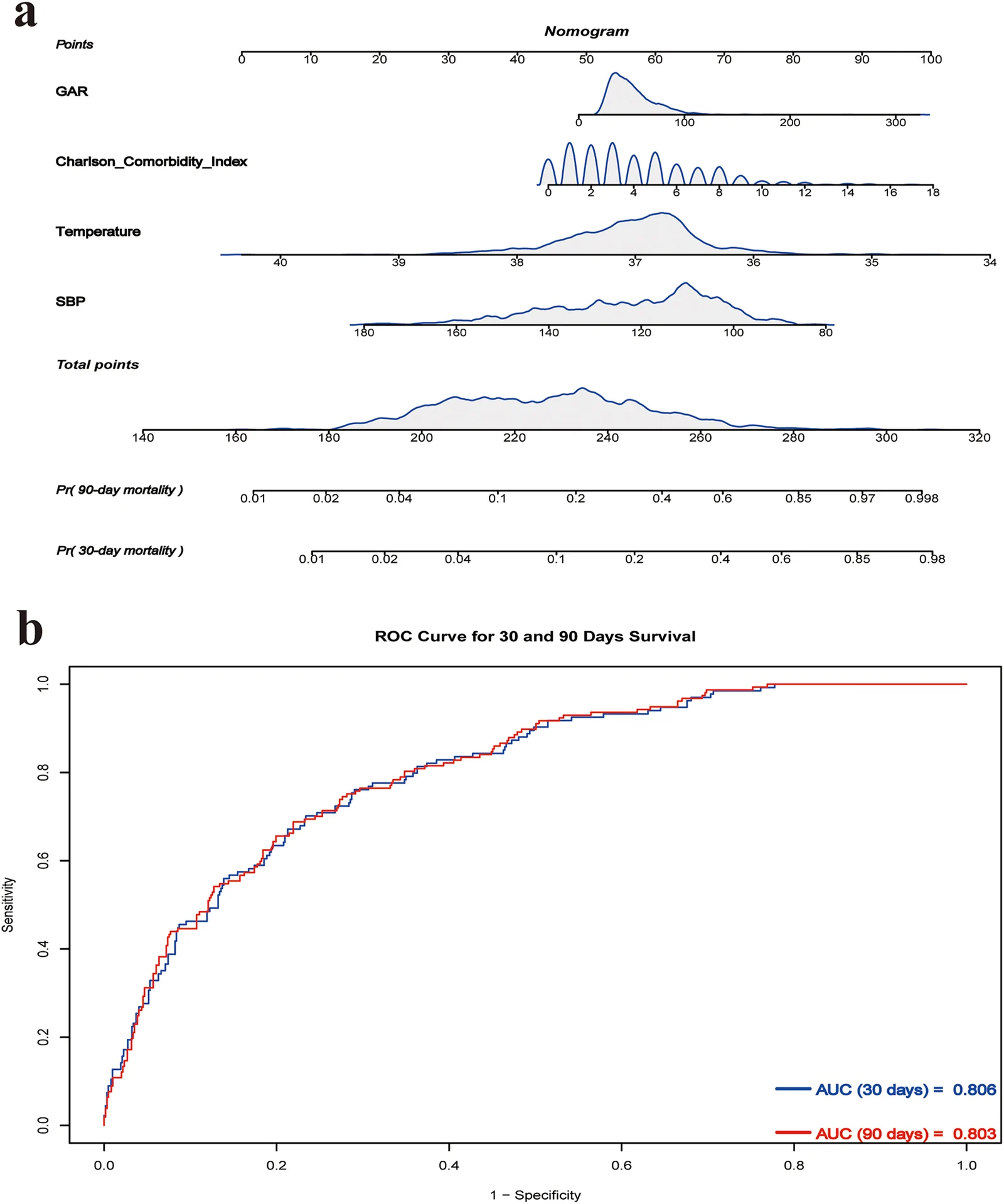
Nomogram construction and discrimination for 30-day and 90-day mortality prediction. (a) Nomogram for predicting 30-day and 90-day mortality, (b) ROC curves for 30-day and 90-day mortality predictions.

To further facilitate clinical use, we constructed the nomogram based on the top four variables identified by SHAP: Charlson Comorbidity Index, SBP, temperature, and GAR. The AUC values for 30-day and 90-day mortality predictions were AUC (30 days)=0.806 and AUC (90 days)=0.803, respectively (Figure 8).

### The results of the sensitivity analysis

Sensitivity analyses showed results generally consistent with the main analysis, with the corresponding results presented in the Supplementary Material (Table S4). For 30-day mortality, the association between GAR and mortality remained significant after excluding patients with corticosteroid use, after additional adjustment for liquid balance or AKI, and in the analysis using natural-log-transformed GAR, whereas the associations were attenuated after excluding patients with insulin therapy, dextrose-containing fluids, or albumin infusion. For 90-day mortality, GAR remained significantly associated with increased mortality in all sensitivity analyses. The baseline characteristics of included and excluded patients are presented in the Supplementary Material (Table S5). Several variables differed between the two groups, including age, GCS, CCI, vital signs, selected laboratory parameters, ERCP, octreotide use, mechanical ventilation, insulin therapy, liquid balance, and 90-day hospital mortality. However, sex, race, major comorbidities, AKI, sepsis, vasopressor use, albumin infusion, dextrose-containing fluid use, corticosteroid use, hospital mortality, and ICU mortality were generally comparable. In addition, major disease severity scores, including SOFA, APS III, and OASIS, were comparable between the two groups.

## Discussion

The study demonstrates a significant association between GAR and all-cause mortality among ICU patients diagnosed with acute pancreatitis. GAR had the highest AUC in ROC analysis for both 30-day and 90-day mortality, surpassing albumin, SOFA, and glucose. Multivariable Cox regression showed that higher GAR was associated with an increased risk of mortality, and RCS analysis confirmed a nonlinear relationship. The Boruta algorithm identified GAR as an important variable, and LightGBM was selected as the best predictive model, with SHAP analysis highlighting GAR as the most important variable.

Compared to previous studies, Zhen et al., Wang et al., and Tang et al. established the prognostic value of GAR in patients with ST-elevation myocardial infarction [11], urinary tract infections after hip surgery [15], and pneumonia [10], respectively. Our study yielded similar findings, showing that elevated GAR is associated with adverse outcomes. However, prior studies on mortality in pancreatitis have primarily focused on broader patient populations, often overlooking the unique challenges faced by ICU patients with acute pancreatitis. These critically ill patients are prone to stress-induced hyperglycemia and rapid metabolic depletion, which significantly impact their nutritional status. By integrating GAR, a marker indicative of glucose fluctuations and nutritional status, our study bridges this gap and underscores its value in predicting all-cause mortality within this high-risk population.

The potential biological mechanisms underlying the association between GAR and mortality remain speculative and should be considered hypothesis-generating. GAR integrates glucose and albumin levels and may therefore reflect the combined effects of metabolic stress, systemic inflammation, nutritional depletion, and organ dysfunction in ICU patients with acute pancreatitis. Hyperglycemia has been associated with oxidative stress, endothelial dysfunction, mitochondrial injury, and impaired immune responses [16], [17], [18], [19]. Acute glucose fluctuations and hypoalbuminemia further exacerbate endothelial injury [20], worsening pancreatic microcirculatory dysfunction. This injury releases pro-inflammatory mediators that damage distant organs such as the lungs [21], kidneys [22], and gut [23]. This promotes bacterial translocation and endotoxemia, further escalating systemic inflammation. Systemically, GAR triggers a pathological cascade of immune dysfunction, initially activating Systemic Inflammatory Response Syndrome [24], which reduces organ perfusion and accelerates multi-organ dysfunction. Prolonged inflammation and nutritional depletion exhaust immune reserves, ultimately leading to immunoparalysis and increasing the risk of secondary infections [25], particularly infected pancreatic necrosis, which remains a primary cause of death in severe acute pancreatitis [26]. These processes may partly explain why elevated GAR is associated with adverse outcomes in this critically ill population.

Our study highlights the clinical importance of GAR as a simple and easily calculable marker, reflecting the interplay between glucose fluctuations and hypoalbuminemia in acute pancreatitis. This simplicity makes GAR an accessible tool for clinicians, enabling rapid risk stratification and early intervention, especially in critically ill patients. Elevated GAR not only serves as a predictor of adverse outcomes but also highlights potential therapeutic avenues. For instance, the use of GLP-1 receptor agonists presents a novel approach, offering dual benefits of anti-inflammatory effects and glucose regulation [27], 28], which could alleviate pancreatic damage and improve outcomes. Additionally, GAR-based early intervention strategies could be complemented by dynamic glucose management, early albumin supplementation, and antioxidant therapies, such as N-acetylcysteine and glutathione [29]. These approaches, aimed at stabilizing metabolic imbalances, hold promise in preventing organ failure and reducing mortality. Furthermore, advancing our mechanistic understanding of GAR could pave the way for targeted therapies that address pancreatic microcirculation dysfunction, oxidative stress, and immune response modulation, ultimately offering new opportunities to enhance outcomes in critically ill patients.

This study has several limitations. First, due to its retrospective design based on the MIMIC-IV database, selection bias and residual confounding cannot be fully excluded. In particular, patients with missing admission glucose or albumin values were excluded from the analysis, which may have introduced potential selection bias. Although sensitivity analyses were performed to assess the robustness of the findings, the missingness of glucose or albumin measurements may not have been completely random and may have been related to clinical testing patterns, physician decision-making, or illness severity. Although multiple covariates were adjusted for in the multivariable analyses, some potential confounders before ICU admission and during treatment may not have been completely captured. Second, ICU patients with acute pancreatitis represent a heterogeneous population with different etiologies and comorbidities, such as biliary or alcoholic pancreatitis, diabetes, and acute kidney injury. These factors may affect disease progression and clinical outcomes, and the prognostic value of GAR may differ across subgroups. In addition, some dynamic clinical variables, such as oxygenation status, cardiac function, and changes in treatment strategies, were not fully captured in this analysis. Finally, although the highest GAR quartile was associated with a markedly increased risk of 30-day mortality, the wide confidence interval indicates uncertainty regarding the precise magnitude of this association. This may be partly related to the limited number of events in some GAR quartiles, sparse-event bias, or residual confounding. Therefore, this finding should be interpreted as evidence of an association rather than as a precise estimate of risk magnitude. Future prospective and multicenter studies with dynamic monitoring are needed to further validate the prognostic value of GAR in this population.

## Conclusions

In conclusion, GAR may serve as a simple and clinically accessible biomarker for mortality risk stratification in ICU patients with acute pancreatitis. Elevated GAR was independently associated with increased mortality risk and showed better prognostic performance than albumin, glucose, and SOFA score. These findings suggest that GAR may provide additional value for early risk assessment in this critically ill population.

## Supplementary Material

Supplementary Material

## References

[1] Głuszek S, Adamus-Białek W, Chrapek M, Dziuba A, Dulębska J, Kozieł D (2024). Genetic variability in the CPA1 gene and its impact on acute pancreatitis risk: new insights from a large-scale study. Int J Mol Sci.

[2] Yang Y, Guo C, Gu Z, Hua J, Zhang J, Qian S (2022). The global burden of appendicitis in 204 countries and territories from 1990 to 2019. Clin Epidemiol.

[3] Glaubitz J, Asgarbeik S, Lange R, Mazloum H, Elsheikh H, Weiss FU (2023). Immune response mechanisms in acute and chronic pancreatitis: strategies for therapeutic intervention. Front Immunol.

[4] Baron TH, DiMaio CJ, Wang AY, Morgan KA (2020). American Gastroenterological Association clinical practice update: management of pancreatic necrosis. Gastroenterology.

[5] Vege SS, DiMagno MJ, Forsmark CE, Martel M, Barkun AN (2018). Initial medical treatment of acute pancreatitis: American Gastroenterological Association Institute technical review. Gastroenterology.

[6] Schepers NJ, Bakker OJ, Besselink MG, Ahmed Ali U, Bollen TL, Gooszen HG (2019). Impact of characteristics of organ failure and infected necrosis on mortality in necrotising pancreatitis. Gut.

[7] Liu Q, Zheng HL, Wu MM, Wang QZ, Yan SJ, Wang M (2022). Association between lactate-to-albumin ratio and 28-days all-cause mortality in patients with acute pancreatitis: a retrospective analysis of the MIMIC-IV database. Front Immunol.

[8] O’Connell RM, Boland MR, O’Driscoll J, Salih A, Arumugasamy M, Walsh TN (2018). Red cell distribution width and neutrophil to lymphocyte ratio as predictors of outcomes in acute pancreatitis: a retrospective cohort study. Int J Surg.

[9] Cho SK, Jung S, Lee KJ, Kim JW (2018). Neutrophil to lymphocyte ratio and platelet to lymphocyte ratio can predict the severity of gallstone pancreatitis. BMC Gastroenterol.

[10] Tang W, Ni X, Yao W, Wang W, Li Y, Lv Q (2024). Glucose-albumin ratio (GAR) as a novel biomarker for predicting postoperative pneumonia (POP) in older adults with hip fractures. Sci Rep.

[11] Zhen C, Chen W, Chen W, Fan H, Lin Z, Zeng L (2023). Association between admission-blood-glucose-to-albumin ratio and clinical outcomes in patients with ST-elevation myocardial infarction undergoing percutaneous coronary intervention. Front Cardiovasc Med.

[12] Zhang Y, Yin X, Liu T, Ji W, Wang G (2024). Association between the stress hyperglycemia ratio and mortality in patients with acute ischemic stroke. Sci Rep.

[13] Lee ZY, Yap CSL, Hasan MS, Engkasan JP, Barakatun-Nisak MY, Day AG (2021). The effect of higher versus lower protein delivery in critically ill patients: a systematic review and meta-analysis of randomized controlled trials. Crit Care.

[14] Wang S, Lin X, Zhu C, Dong Y, Guo Y, Xie Z (2023). Association between nonalcoholic fatty liver disease and increased glucose-to-albumin ratio in adults without diabetes. Front Endocrinol.

[15] Wang W, Tang W, Yao W, Lv Q, Ding W (2024). Glucose-albumin ratio (GAR) as a novel biomarker of postoperative urinary tract infection in elderly hip fracture patients. Front Med.

[16] Cham ED, Peng TI, Jou MJ (2024). Pathological role of high sugar in mitochondrial respiratory chain defect-augmented mitochondrial stress. Biology.

[17] Sastre J, Pérez S, Sabater L, Rius-Pérez S (2025). Redox signaling in the pancreas in health and disease. Physiol Rev.

[18] He J, Ma M, Li D, Wang K, Wang Q, Li Q (2021). Sulfiredoxin-1 attenuates injury and inflammation in acute pancreatitis through the ROS/ER stress/Cathepsin B axis. Cell Death Dis.

[19] Xiang H, Guo F, Tao X, Zhou Q, Xia S, Deng D (2021). Pancreatic ductal deletion of S100A9 alleviates acute pancreatitis by targeting VNN1-mediated ROS release to inhibit NLRP3 activation. Theranostics.

[20] Adeshara K, Di Marco E, Bordino M, Gordin D, Bernardi L, Cooper ME (2024). Altered oxidant and antioxidant levels are associated with vascular stiffness and diabetic kidney disease in type 1 diabetes after exposure to acute and chronic hyperglycemia. Cardiovasc Diabetol.

[21] Liu D, Wen L, Wang Z, Hai Y, Yang D, Zhang Y (2022). The mechanism of lung and intestinal injury in acute pancreatitis: a review. Front Med.

[22] Zhang X, Ye B, Mao W, Liu L, Li G, Zhou J (2022). Major adverse kidney events within 30 days in patients with acute pancreatitis: a tertiary-center cohort study. HPB (Oxford).

[23] Zhang C, Li G, Lu T, Liu L, Sui Y, Bai R (2023). The interaction of microbiome and pancreas in acute pancreatitis. Biomolecules.

[24] Prokazyuk A, Tlemissov A, Zhanaspayev M, Aubakirova S, Mussabekov A (2024). Development and validation of a machine learning-based model to assess probability of systemic inflammatory response syndrome in patients with severe multiple traumas. BMC Med Inf Decis Making.

[25] Kothari A, Singh V, Nath UK, Kumar S, Rai V, Kaushal K (2020). Immune dysfunction and multiple treatment modalities for the SARS-CoV-2 pandemic: races of uncontrolled running sweat?. Biology.

[26] Leppäniemi A, Tolonen M, Tarasconi A, Segovia-Lohse H, Gamberini E, Kirkpatrick AW (2019). 2019 WSES guidelines for the management of severe acute pancreatitis. World J Emerg Surg.

[27] Yao H, Zhang A, Li D, Wu Y, Wang CZ, Wan JY (2024). Comparative effectiveness of GLP-1 receptor agonists on glycaemic control, body weight, and lipid profile for type 2 diabetes: systematic review and network meta-analysis. Bmj.

[28] Zhang X, Wang Y, Cai Z, Wan Z, Aihemaiti Y, Tu H (2023). A gonadal gap junction INX-14/Notch GLP-1 signaling axis suppresses gut defense through an intestinal lysosome pathway. Front Immunol.

[29] Sahasrabudhe SA, Terluk MR, Kartha RV (2023). N-acetylcysteine pharmacology and applications in rare diseases-repurposing an old antioxidant. Antioxidants.

