# Supplementary material for: Glucose-to-albumin ratio and mortality in ICU patients with acute pancreatitis
Source: Open Med (Wars). 2026 Aug 31;21(1):20261511. doi: 10.1515/med-2026-1511 (PMC13524130; doi:10.1515/med-2026-1511)
Supplement: Supplementary file 1 [file j_med-2026-1511_suppl_001.docx]

***Supplementary Material***

Table S1 Missing values in variables.

Table S2 Univariable cox regression analysis for 30-day in-hospital all-cause mortality.

Table S3 Characteristics of patient in training set and validation set

Table S4 Results of sensitivity analyses

Table S5 Baseline characteristics of included and excluded patients

Fig. S1 Proportional hazards assumption test for the Cox regression models: (a) 30-day all-cause mortality; (b) 90-day all-cause mortality.

Fig. S2 (a) ROC curve for 30-day mortality, (b) ROC curve for 90-day mortality, (c) ROC curve for in-hospital mortality, (d) ROC curve for ICU mortality.

Fig. S3 (a) Calibration curves for multiple models in the validation Set. (b) Decision curves for multiple models in the validation set.

**Table S1: Missing Values in Variables**

| **Variable** | **Missing Percentage (%)** |
| --- | --- |
| PT | 7.43 |
| INR | 7.43 |
| ALT | 4.24 |
| AST | 3.58 |
| Temperature | 0.66 |
| Calcium | 0.53 |
| SBP | 0.4 |
| SOFA | 0.27 |
| GCS | 0.27 |
| BUN | 0.27 |
| Heart Rate | 0.13 |
| Respiratory Rate | 0.13 |
| SPO2 | 0.13 |
| WBC | 0.13 |

**Table S2: Univariable Cox Regression Analysis for 30-Day In-Hospital Mortality.**

| **Variables** | **β** | **S.E** | **Z** | **P** | **HR (95%CI)** |
| --- | --- | --- | --- | --- | --- |
|  |  |  |  |  |  |
| Gender |  |  |  |  |  |
| female |  |  |  |  | 1.00 (Reference) |
| male | 0.19 | 0.21 | 0.91 | 0.361 | 1.21 (0.81 ~ 1.80) |
| Race |  |  |  |  |  |
| white |  |  |  |  | 1.00 (Reference) |
| other | 0.43 | 0.20 | 2.12 | **0.034** | 1.54 (1.03 ~ 2.29) |
| Age | 0.04 | 0.01 | 6.17 | **<.001** | 1.04 (1.03 ~ 1.05) |
| SOFA | 0.18 | 0.03 | 5.95 | **<.001** | 1.20 (1.13 ~ 1.27) |
| Charlson Comorbidity Index | 0.19 | 0.03 | 7.30 | **<.001** | 1.21 (1.15 ~ 1.28) |
| SBP | -0.05 | 0.01 | -6.93 | **<.001** | 0.95 (0.93 ~ 0.96) |
| RBC | -0.29 | 0.15 | -1.95 | 0.051 | 0.75 (0.56 ~ 1.00) |
| WBC | 0.04 | 0.01 | 5.54 | **<.001** | 1.04 (1.03 ~ 1.06) |
| ALT | 0.01 | 0.00 | 4.15 | **<.001** | 1.01 (1.01 ~ 1.01) |
| AST | 0.01 | 0.00 | 8.02 | **<.001** | 1.01 (1.01 ~ 1.01) |
| Anion gap | 0.15 | 0.02 | 7.83 | **<.001** | 1.16 (1.12 ~ 1.20) |
| GAR | 0.02 | 0.00 | 5.38 | **<.001** | 1.02 (1.01 ~ 1.03) |
| Malignant Cancer |  |  |  |  |  |
| no |  |  |  |  | 1.00 (Reference) |
| yes | 0.87 | 0.26 | 3.33 | **<.001** | 2.38 (1.43 ~ 3.96) |
| Diabetes |  |  |  |  |  |
| no |  |  |  |  | 1.00 (Reference) |
| yes | 0.19 | 0.21 | 0.91 | 0.360 | 1.21 (0.80 ~ 1.82) |
| Hypertension |  |  |  |  |  |
| no |  |  |  |  | 1.00 (Reference) |
| yes | 0.03 | 0.21 | 0.16 | 0.874 | 1.03 (0.68 ~ 1.56) |
| Sepsis |  |  |  |  |  |
| no |  |  |  |  | 1.00 (Reference) |
| yes | 1.04 | 0.26 | 4.01 | **<.001** | 2.84 (1.70 ~ 4.72) |
| Ercp |  |  |  |  |  |
| no |  |  |  |  | 1.00 (Reference) |
| yes | -0.43 | 0.59 | -0.74 | 0.460 | 0.65 (0.21 ~ 2.05) |
| Norepinephrine |  |  |  |  |  |
| no |  |  |  |  | 1.00 (Reference) |
| yes | 1.59 | 0.20 | 7.78 | **<.001** | 4.90 (3.28 ~ 7.31) |
| Octreotide |  |  |  |  |  |
| no |  |  |  |  | 1.00 (Reference) |
| yes | 0.65 | 0.37 | 1.76 | 0.078 | 1.91 (0.93 ~ 3.94) |
| Mechanical Ventilation |  |  |  |  |  |
| no |  |  |  |  | 1.00 (Reference) |
| yes | 0.51 | 0.28 | 1.84 | 0.066 | 1.67 (0.97 ~ 2.90) |
| CRRT |  |  |  |  |  |
| no |  |  |  |  | 1.00 (Reference) |
| yes | 1.82 | 0.21 | 8.87 | **<.001** | 6.18 (4.13 ~ 9.25) |
| Heart Rate | -0.01 | 0.01 | -0.87 | 0.383 | 0.99 (0.98 ~ 1.01) |
| Respiratory Rate | 0.09 | 0.02 | 4.24 | **<.001** | 1.09 (1.05 ~ 1.14) |
| Chronic Pulmonary Disease |  |  |  |  |  |
| no |  |  |  |  | 1.00 (Reference) |
| yes | 0.14 | 0.24 | 0.58 | 0.564 | 1.15 (0.72 ~ 1.83) |
| Platelet | -0.00 | 0.00 | -1.11 | 0.266 | 1.00 (1.00 ~ 1.00) |
| HR: Hazard Ratio, CI: Confidence Interval |  |  |  |  |  |

**Table S3 Characteristics of patient in training set and validation set**

| **Characteristic** | **Overall**  N = 754 | **training**  N = 527 | **validation**  N = 227 | **p-value** |
| --- | --- | --- | --- | --- |
| Age | 60.34 (47.47, 74.06) | 60.91 (48.19, 73.87) | 59.64 (44.83, 75.90) | 0.481 |
| SOFA | 1.00 (0.00, 3.00) | 1.00 (0.00, 3.00) | 1.00 (0.00, 3.00) | 0.616 |
| Charlson comorbidity index | 3.00 (2.00, 6.00) | 3.00 (2.00, 6.00) | 3.00 (1.00, 6.00) | 0.798 |
| SBP | 119.02 (108.51, 134.17) | 119.00 (107.59, 134.18) | 119.30 (109.74, 133.92) | 0.512 |
| Respiratory rate | 20.88 (17.79, 24.16) | 20.96 (17.69, 24.21) | 20.42 (17.94, 23.76) | 0.673 |
| Temperature | 36.97 (36.69, 37.35) | 36.97 (36.67, 37.33) | 36.99 (36.73, 37.38) | 0.149 |
| SpO2 | 96.11 (94.85, 97.43) | 96.11 (94.84, 97.33) | 96.11 (94.88, 97.52) | 0.249 |
| Malignant cancer | 68 (9%) | 49 (9%) | 19 (8%) | 0.683 |
| WBC | 12.60 (8.70, 17.80) | 12.83 (9.03, 17.94) | 11.97 (8.35, 17.33) | 0.246 |
| Platelet | 185.00 (128.00, 260.00) | 186.33 (128.50, 260.33) | 180.00 (122.50, 259.67) | 0.493 |
| Sodium | 138.00 (135.20, 140.75) | 138.00 (135.00, 140.67) | 138.25 (135.60, 141.09) | 0.110 |
| Potassium | 4.00 (3.70, 4.39) | 4.00 (3.70, 4.40) | 4.00 (3.68, 4.35) | 0.561 |
| GAR | 25.22 (19.18, 33.77) | 25.26 (19.42, 33.83) | 25.21 (18.53, 33.48) | 0.665 |
| ALT | 48.88 (24.00, 137.00) | 47.33 (23.67, 134.00) | 58.00 (25.33, 164.50) | 0.322 |
| AST | 69.00 (34.50, 150.33) | 68.50 (34.50, 143.75) | 70.00 (33.50, 179.67) | 0.575 |
| PT | 14.23 (12.93, 16.45) | 14.35 (12.96, 16.53) | 14.10 (12.85, 16.30) | 0.668 |
| INR | 1.30 (1.15, 1.50) | 1.30 (1.17, 1.50) | 1.30 (1.15, 1.50) | 0.842 |
| Anion gap | 14.50 (12.25, 17.00) | 14.33 (12.00, 16.60) | 14.75 (12.25, 17.33) | 0.226 |
| BUN | 19.17 (11.67, 35.50) | 20.00 (12.00, 35.67) | 18.00 (11.00, 33.67) | 0.306 |
| Creatinine | 1.00 (0.70, 1.90) | 0.98 (0.70, 1.92) | 1.03 (0.70, 1.90) | 0.666 |
| Norepinephrine used, n (%) | 198 (26%) | 139 (26%) | 59 (26%) | 0.912 |
| Dopamine used, n (%) | 20 (3%) | 14 (3%) | 6 (3%) | 0.992 |
| CRRT | 87 (12%) | 58 (11%) | 29 (13%) | 0.485 |
| 30-day hospital Mortality (%) | 100 (13%) | 70 (13%) | 30 (13%) | 0.980 |

**Table S4 Results of sensitivity analyses**

| **Outcome** | **Sensitivity analysis** | **N** | **Events** | **HR** | **95% CI** | **p-value** |
| --- | --- | --- | --- | --- | --- | --- |
| 30-day outcome | Main analysis | 754 | 100 | 1.02 | 1.01, 1.03 | 0.002 |
| 30-day outcome | Excluding insulin therapy | 403 | 56 | 1.02 | 1.00, 1.04 | 0.051 |
| 30-day outcome | Excluding dextrose-containing fluids | 218 | 34 | 1.01 | 0.98, 1.03 | 0.598 |
| 30-day outcome | Excluding albumin infusion | 478 | 62 | 1.01 | 1.00, 1.02 | 0.094 |
| 30-day outcome | Excluding corticosteroid use | 738 | 98 | 1.02 | 1.01, 1.03 | <0.001 |
| 30-day outcome | Additional adjustment for liquid balance | 754 | 100 | 1.02 | 1.01, 1.03 | 0.002 |
| 30-day outcome | Additional adjustment for AKI | 754 | 100 | 1.02 | 1.01, 1.03 | 0.003 |
| 30-day outcome | Log-transformed GAR | 754 | 100 | 2.84 | 1.66, 4.86 | <0.001 |
| 90-day outcome | Main analysis | 754 | 157 | 1.02 | 1.01, 1.03 | <0.001 |
| 90-day outcome | Excluding insulin therapy | 403 | 89 | 1.03 | 1.01, 1.04 | <0.001 |
| 90-day outcome | Excluding dextrose-containing fluids | 218 | 53 | 1.02 | 1.00, 1.04 | 0.048 |
| 90-day outcome | Excluding albumin infusion | 478 | 100 | 1.02 | 1.01, 1.03 | <0.001 |
| 90-day outcome | Excluding corticosteroid use | 738 | 154 | 1.02 | 1.01, 1.03 | <0.001 |
| 90-day outcome | Additional adjustment for liquid balance | 754 | 157 | 1.02 | 1.01, 1.03 | <0.001 |
| 90-day outcome | Additional adjustment for AKI | 754 | 157 | 1.02 | 1.01, 1.03 | <0.001 |
| 90-day outcome | Log-transformed GAR | 754 | 157 | 2.97 | 1.95, 4.53 | <0.001 |
| Abbreviations: AKI = acute kidney injury; CI = confidence interval; GAR = glucose-to-albumin ratio; HR = hazard ratio. Log-transformed GAR refers to natural-log-transformed GAR. N and Events refer to the complete-case sample used in each Cox model. | | | | | | |

**Table S5 Baseline characteristics of included and excluded patients**

| **Characteristic** | **Included**  N = 754 | **Excluded**  N = 526 | **p-value**^1^ |
| --- | --- | --- | --- |
| Age(years) | 60.34 (47.47, 74.06) | 58.13 (45.88, 70.97) | 0.034 |
| Gender, male (%) | 427 (57%) | 308 (59%) | 0.493 |
| Race, n(%) |  |  | 0.965 |
| White | 508 (67%) | 355 (67%) |  |
| Black | 246 (33%) | 171 (33%) |  |
| SOFA | 1.00 (0.00, 3.00) | 1.00 (0.00, 4.00) | 0.729 |
| APS III | 48.00 (36.00, 68.00) | 47.00 (33.00, 64.00) | 0.168 |
| OASIS | 15.00 (15.00, 15.00) | 15.00 (15.00, 15.00) | 0.881 |
| GCS | 33.00 (26.00, 40.00) | 31.00 (24.00, 38.00) | <0.001 |
| CCI | 3.00 (2.00, 6.00) | 4.00 (2.00, 6.00) | <0.001 |
| Heart rate (bpm) | 95.00 (83.22, 107.38) | 89.01 (77.86, 104.37) | <0.001 |
| SBP (mmHg) | 119.02 (108.51, 134.17) | 118.11 (107.08, 132.49) | 0.308 |
| Respiratory rate (bpm) | 20.88 (17.79, 24.16) | 19.53 (16.97, 22.75) | <0.001 |
| Temperature (°C) | 36.97 (36.69, 37.35) | 36.84 (36.64, 37.14) | <0.001 |
| SpO2 (%) | 96.11 (94.85, 97.43) | 96.38 (94.92, 97.82) | 0.037 |
| Chronic pulmonary disease, n (%) | 157 (21%) | 103 (20%) | 0.587 |
| Malignant cancer, n (%) | 68 (9%) | 42 (8%) | 0.516 |
| Diabetes, n (%) | 241 (32%) | 160 (30%) | 0.558 |
| Hypertension, n (%) | 253 (34%) | 157 (30%) | 0.162 |
| Aki, n (%) | 525 (70%) | 348 (66%) | 0.190 |
| Sepsis, n (%) | 476 (63%) | 318 (60%) | 0.332 |
| RBC (10^9^/L) | 3.55 (3.12, 4.05) | 3.42 (2.91, 3.91) | <0.001 |
| WBC (10^9^/L) | 12.60 (8.70, 17.80) | 11.36 (7.75, 15.65) | <0.001 |
| Platelet (10^9^/L) | 185.00 (128.00, 260.00) | 166.17 (107.60, 232.13) | <0.001 |
| Sodium (mmol/L) | 138.00 (135.20, 140.75) | 137.50 (135.00, 140.43) | 0.292 |
| Potassium (mmol/L) | 4.00 (3.70, 4.39) | 3.99 (3.70, 4.44) | 0.850 |
| Calcium (mg/dL) | 7.93 (7.45, 8.45) | 8.20 (7.70, 8.65) | <0.001 |
| ALT (U/L) | 48.88 (24.00, 137.00) | 49.83 (24.00, 144.00) | 0.818 |
| AST (U/L) | 69.00 (34.50, 150.33) | 74.67 (36.00, 186.00) | 0.137 |
| PT (S) | 14.23 (12.93, 16.45) | 14.55 (12.97, 18.10) | 0.038 |
| INR | 1.30 (1.15, 1.50) | 1.30 (1.17, 1.67) | 0.037 |
| Anion gap (m q/L) | 14.50 (12.25, 17.00) | 14.50 (12.33, 17.50) | 0.249 |
| BUN (mg/dL) | 19.17 (11.67, 35.50) | 18.65 (11.00, 37.00) | 0.921 |
| Creatinine (mg/dL) | 1.00 (0.70, 1.90) | 1.09 (0.70, 1.95) | 0.263 |
| ERCP, n (%) | 34 (5%) | 11 (2%) | 0.021 |
| Norepinephrine, n (%) | 198 (26%) | 139 (26%) | 0.947 |
| Dopamine, n (%) | 20 (3%) | 9 (2%) | 0.265 |
| Octreotide, n (%) | 35 (5%) | 90 (17%) | <0.001 |
| MV, n (%) | 587 (78%) | 384 (73%) | 0.046 |
| CRRT, n (%) | 87 (12%) | 80 (15%) | 0.055 |
| Albumin infusion, n (%) | 276 (37%) | 191 (36%) | 0.915 |
| Dextrose fluid, n (%) | 536 (71%) | 357 (68%) | 0.218 |
| Corticosteroid, n (%) | 16 (2%) | 16 (3%) | 0.300 |
| Insulin, n (%) | 351 (47%) | 210 (40%) | 0.019 |
| Liquid balance, (mL) | 3,857.72 (1,312.35, 6,125.33) | 3,397.17 (948.42, 4,879.71) | <0.001 |
| Hospital Mortality (%) | 108 (14%) | 91 (17%) | 0.148 |
| ICU Mortality (%) | 70 (9%) | 53 (10%) | 0.636 |
| Los of Hospital （day） | 12.59 (6.59, 21.96) | 11.07 (5.62, 22.22) | 0.168 |
| Los of ICU (day) | 108 (14%) | 91 (17%) | 0.148 |
| 30-day hospital Mortality (%) | 70 (9%) | 53 (10%) | 0.636 |
| 90-day hospital Mortality (%) | 100 (13%) | 92 (17%) | 0.037 |
| Hospital Mortality (%) | 157 (21%) | 119 (23%) | 0.441 |
| Missing GAR, n (%) | 0 (0%) | 382 (73%) | <0.001 |

**Fig. S1 Proportional hazards assumption test for the Cox regression models: (a) 30-day all-cause mortality; (b) 90-day all-cause mortality.**

**Fig. S2 (a) ROC curve for 30-day mortality, (b) ROC curve for 90-day mortality, (c) ROC curve for in-hospital mortality, (d) ROC curve for ICU mortality.**

**Fig. S3 (a) Calibration curves for multiple models in the validation Set. (b) Decision curves for multiple models in the validation set.**
